# Inhibition of the Rho/Rho kinase pathway prevents lipopolysaccharide-induced hyperalgesia and the release of TNF-α and IL-1β in the mouse spinal cord

**DOI:** 10.1038/srep14553

**Published:** 2015-09-29

**Authors:** Cunjin Wang, Siyuan Song, Yang Zhang, Yali Ge, Xiangzhi Fang, Tianfeng Huang, Jin Du, Ju Gao

**Affiliations:** 1 Clinical Medical College of Yangzhou University & Department of Anesthesiology, Subei People’s Hospital of Jiangsu Province, Yangzhou, China; 2 Jiangsu Key Laboratory of Anesthesiology & Jiangsu Key Laboratory of Anesthesia and Analgesia Application Technology, Xuzhou Medical College, Xuzhou, China

## Abstract

Administration of lipopolysaccharide (LPS) by various routes produces profound inflammatory pain hypersensitivity. However, the molecular events that induce this response remain largely uncharacterized. In the present study, we sought to elucidate the role of the Rho/Rho kinase (ROCK) pathway in the release of tumor necrosis factor-α (TNF-α) and interleukin 1β (IL-1β) following injection of LPS into the mouse paw, which is associated with nociceptive behavior. The spinal cord of LPS-treated mice showed increased active GTP-bound RhoA and upregulation of ROCK2 and c-fos compared to the normal saline group. Furthermore, the inflammation-related cytokines TNF-α and IL-1β were markedly increased in the spinal dorsal horn after intraplantar injection of LPS. However, the latter effects were prevented by prophylactic intrathecal administration of the Rho inhibitor (C3 exoenzyme) or the ROCK inhibitor (Y27632). Collectively, our results suggest that the Rho/ROCK signaling pathway plays a critical role in LPS-induced inflammatory pain and that this pathway is coincident with the release of the pro-nociceptive cytokines TNF-α and IL-1β, which produces hyperalgesia.

Inflammatory pain is a common symptom of a variety of inflammatory diseases. It can be triggered by different stimuli, such as infection, radiation, antigen and trauma. Because the progression of inflammatory pain influences daily life and may affect the prognosis of patients, it is very important to prevent the development of inflammatory pain by treating it at an early stage. However, the mechanisms underlying inflammatory pain are not completely understood; therefore, effective treatment remains a challenge. Among the mechanisms involved in the genesis of inflammatory pain, the sensitization of pain signaling pathways is a central event.

Lipopolysaccharide (LPS) is a well-recognized TLR4 agonist that is a component of gram-negative bacterial walls, and inflammation induced by LPS has been used as a model representing gram-negative bacteria-induced inflammation^1^,^2^,^3^. In addition, LPS can be used to assess the involvement of TLR4 in inducing acute and chronic pain^4^,^5^. For instance, intraplantar or intrathecal injection of LPS induces a dose-dependent mechanical inflammatory hyperalgesia and can be used as a model of inflammatory pain^4^,^6^,^7^. Here we use this model to elucidate the role of the spinal Rho/ROCK signaling pathway in the pathogenesis of LPS-induced inflammatory hyperalgesia.

RhoA is a member of a family of small molecular G proteins, which are involved in many cellular functions including cytoskeletal rearrangement, cell motility, phagocytosis, intracellular trafficking, transcriptional regulation, and cell growth and development. Small molecular G proteins are part of the larger Ras superfamily of monomeric GTPases^8^,^9^. These small molecular G proteins are thought to act as molecular switches integrating signals from the extracellular environment. They cycle between two conformational states (active GTP-bound and inactive GDP-bound) by hydrolyzing GTP to GDP^10^. Several studies using region-specific conditional deletion of the small GTPases during development demonstrate diverse roles for Cdc42, Rac1 and RhoA in embryonic neurogenesis and neuronal maturation. For instance, knockouts of Cdc42 and Rac1 are lethal during embryogenesis, with death occurring by E9.5 11,12. However, pharmacological inhibition of RhoA signaling *in vivo* increases newborn neuron survival^13^. Collectively, these results suggest that Cdc42 and Rac1 are involved in proliferation and dendritic and spine maturation, whereas RhoA may have a “negative” role in neuronal survival and maturation in the central nervous system. Moreover, RhoA has also been shown to play an important role in the formation of long-term potentiation in hippocampal neurons^14^.

Increasing evidence has shown that the synaptic plasticity of dorsal horn neurons contributes to pain hypersensitivity after noxious stimulation^15^,^16^. Additionally, noxious stimulation of the sciatic nerve induces long-term potentiation of C-fiber-evoked field potentials in the spinal dorsal horn and leads to persistent pain^17^. Several intracellular signaling pathway and protein kinase cascades mediate the formation of synaptic plasticity of dorsal horn neurons after noxious stimulation^18^. Recent studies demonstrated that the activation of the spinal RhoA/ROCK signaling pathway plays an important role in the development and maintenance of neuropathic pain^19^,^20^,^21^. For example, intrathecal treatment with mevalonate produced thermal hyperalgesia through the activation of spinal RhoA/ROCK signaling^22^. However, the role of the spinal Rho/ROCK pathway in LPS-induced inflammatory hyperalgesia remains to be elucidated. Thus, we evaluated whether the spinal Rho/ROCK pathway contributes to LPS-induced hyperalgesia. Our results suggest that the Rho/ROCK signaling pathway plays a critical role in LPS-induced inflammatory pain and that this pathway may trigger the release of the pro-nociceptive cytokines TNF-α and IL-1β.

## Results

### LPS treatment induces hyperalgesia and c-fos activation

**Figure 1:**
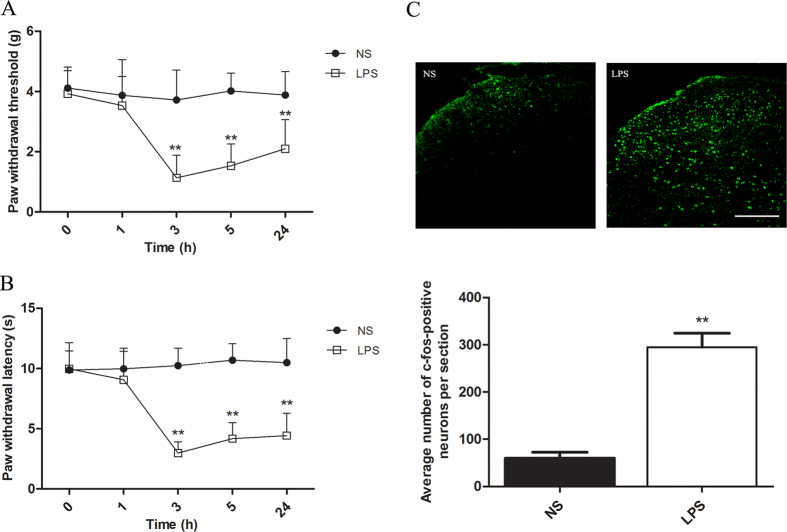
Intraplantar injection of LPS produces behavioral hyperalgesia and spinal neuron activation. (**A**) Intraplantar injection of LPS produces mechanical hyperalgesia. **P < 0.01 vs. normal saline (NS) group by two-way ANOVA; *n *= 8. (**B**) LPS-induces thermal hyperalgesia manifested as a lowered threshold of thermal withdrawal. Eight mice were included in each group. Two-way ANOVA, **P < 0.01 versus NS group. (**C**) Representative immunohistochemical staining and quantitative data of c-fos in the spinal cord of mice. The average number of c-fos-positive neurons per section was calculated for each group. Intraplantar injection of LPS increased spinal c-fos expression. **P < 0.01 compared with NS group by Student’s t test, *n *= 8 mice in each group. Scale bar = 150 μm.

Previous studies have shown that LPS produces hyperalgesia in animals^6^,^23^,^24^. In agreement with these reports, our results show that intraplantar injection of LPS induces mechanical and thermal hyperalgesia in mice that lasts about 24 h or longer after intraplantar injection of LPS (Fig. 1A,B). To further clarify the algesic effect of LPS, we investigated the change in expression of spinal c-fos protein at 3 h after intraplantar injection of LPS, at which time hyperalgesia is most obvious. The expression of c-fos protein, the product of the c-fos immediate early gene, has been shown to correlate with spinal neuronal activation induced by nociceptive stimuli and thus has been used as a marker for neuronal activation in the central nervous system^25^. Our results demonstrate that intraplantar injection of LPS induces a remarkable increase of spinal c-fos protein expression (Fig. 1C), which provides indirect evidence for the activation of neuronal pathways.

### LPS activates the RhoA/ROCK signaling pathway in the spinal dorsal horn

**Figure 2:**
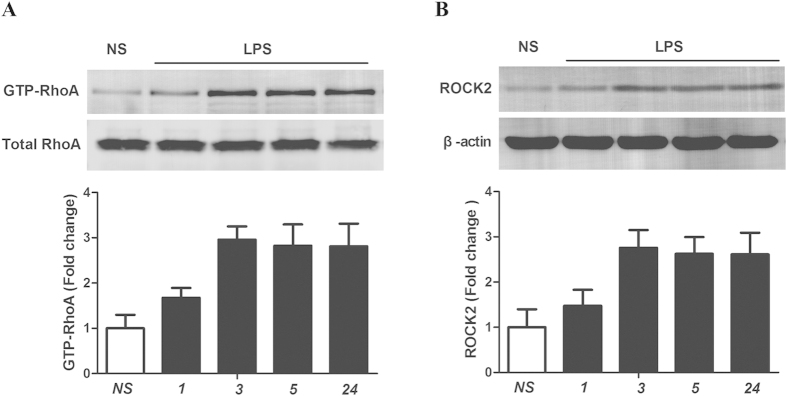
LPS-induced activation of the RhoA/ROCK pathway in the dorsal horn. (**A**) Active GTP–RhoA was assessed by pull-down assay after treatment with NS or at the indicated time points after treatment with LPS. Paw injection of LPS produced a rapid-onset (within 3 h) increase in the expression of GTP-bound RhoA in the dorsal horn. Expression was standardized to the expression of total RhoA in the dorsal horn and normalized to the average expression in NS mice (1.0). A representative experiment is shown above, and quantification is shown below. **P < 0.01 versus NS group by one-way ANOVA; *n *= 4. (**B**) Representative western blot showing the upregulation of ROCK2 following LPS treatment. Normalization and quantification was performed as in panel A. **P < 0.01 versus NS group by one-way ANOVA; *n *= 4.

To investigate the mechanisms of LPS-induced hyperalgesia, we determined whether the Rho pathway may act on the dorsal horn. GTP-bound (active form) RhoA in the dorsal horn was measured using a RhoA activity assay, and total-RhoA was examined by Western blotting. Our results demonstrate that paw injection of LPS produced a rapid-onset (within 3 h) increase in the levels of GTP-bound RhoA in the dorsal horn, while the total-RhoA levels remained unchanged (Fig. 2A). This suggests that RhoA is activated in the dorsal horn after paw injection of LPS. ROCK, the first identified and best characterized effector of RhoA, plays an important role in activating the RhoA pathway. ROCK1 is preferentially expressed in the liver, spleen, kidney and testis, while ROCK2 is predominant in other tissues, especially in the nervous and skeletal muscle^26^. Therefore, we next investigated whether ROCK2 is associated with the changes in the levels of GTP-bound RhoA. Western blot analysis demonstrated that ROCK2 was markedly up-regulated after paw injection of LPS, with a similar timecourse of up-regulation as for RhoA (Fig. 2B). On the basis of these results, the spinal RhoA/ROCK signaling pathway may be involved in the development of inflammatory pain induced by intraplantar injection of LPS.

### LPS evokes release of pro-inflammatory cytokines TNF-α and IL-1β

**Figure 3:**
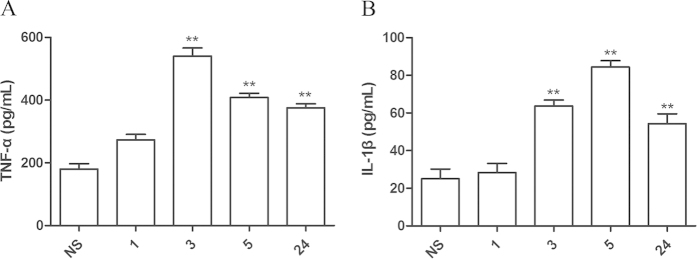
Intraplantar injection of LPS increases the expression of pro-inflammatory cytokines TNF-α and IL-1β in the dorsal horn. Saline (20 μL/paw) or LPS (100 ng/paw) was injected into the hindpaw of mice. At the indicated times, the dorsal half of the 4–5th lumbar spinal cord was collected for (A) TNF-α, (B) IL-1β determination by ELISA. **P < 0.01 compared with NS group by one-way ANOVA; *n *= 8.

TNF-α and IL-1β are important regulators of innate and acquired immune responses and are essential for the generation of pain^27^,^28^. To determine whether LPS activates the release of TNF-α and IL-1β in our model, the levels of these pro-inflammatory cytokines were examined by ELISA. The levels of TNF-α and IL-1β in the dorsal horn were significantly increased in LPS-treated mice (Fig. 3A,B). Interestingly, maximal TNF-α levels were observed at 3 h, whereas the increase in IL-1β was delayed, with a peak at 5 h.

### LPS-induced hyperalgesia and c-fos activation is suppressed by Rho/ROCK signaling antagonists

**Figure 4:**
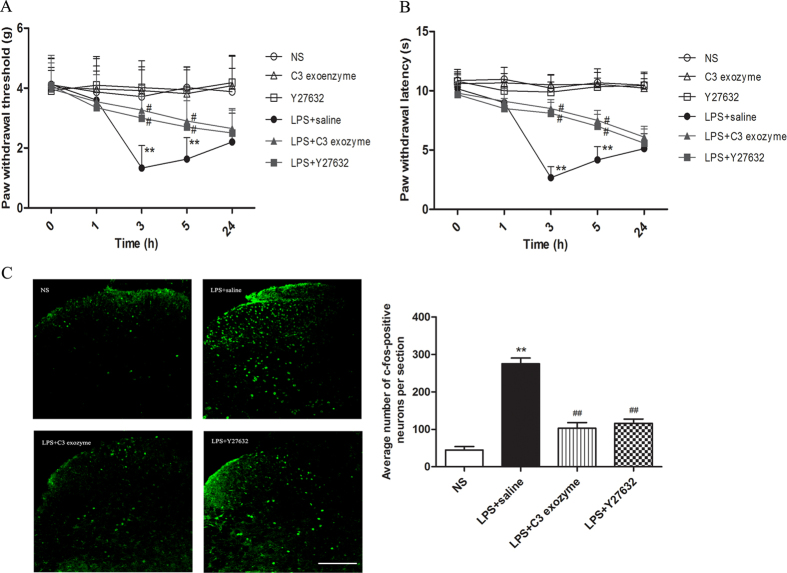
Disruption of the Rho/ROCK pathway attenuates LPS-induced hyperalgesia and spinal neuronal sensitization. (**A**) Pre-treatment with C3 exoenzyme and Y27632 inhibited mechanical hyperalgesia induced by intraplantar injection of LPS. (**B**) Pre-injection of C3 exoenzyme and Y27632 inhibits thermal hyperalgesia induced by intraplantar injection of LPS. Mechanical hyperalgesia and thermal hyperalgesia were evaluated after LPS injection. Two-way ANOVA, **P < 0.01 compared with NS control; #*P *< 0.05 compared with LPS+saline group. *n *= 8 mice in each group. (**C**) The increasing expression of spinal c-fos induced by LPS in mice can be abolished by pre-injection of C3 exoenzyme or Y27632. At 3 h after LPS injection, the 4–5th lumbar spinal cord was collected for c-fos. Representative immunohistochemical staining of c-fos in the spinal cord of mice in the NS group, LPS+saline group, LPS+C3 exoenzyme group and LPS+Y27632 group. Quantitative data indicates the average number of c-fos-positive neurons in the spinal cord sections of mice in each group. One-way ANOVA, **P < 0.01 compared with NS control; ##*P *< 0.01 compared with LPS+saline group. *n *= 8 mice in each group. Scale bar = 150 μm.

To further validate the role of Rho/ROCK pathway activation in LPS-induced pain, we examined nociceptive behavior in the presence of Rho and ROCK antagonists. We administered C3 exoenzyme or Y27632 intrathecally 30 min prior to intraplantar injection of LPS and evaluated pain behavior at the indicated time points after treatment with LPS. Treatment with these antagonists significantly attenuated the hyperalgesia response at 3 and 5 h after intraplantar injection of LPS. In contrast, the pain behavior in naïve mice was not affected by treatment with C3 exoenzyme or Y27632 (Fig. 4 A,B), indicating that C3 exoenzyme or Y27632 improved hyperalgesia but did not affect the normal nociceptive threshold. These results suggest that the Rho/ROCK pathway plays an essential role in the LPS induced hyperalgesia. Given that intraplantar injection of LPS also increases spinal c-fos expression (Fig. 1C), we investigated whether prophylactic intrathecal administration of Rho and ROCK inhibitors could reverse the increase of c-fos in the dorsal horn. Our results demonstrate that pretreatment with C3 exoenzyme or Y27632 30 min before intraplantar injection of LPS prevents the increase of spinal c-fos expression (Fig. 4C). These results further suggest that the Rho/ROCK pathway may be involved in LPS-induced inflammatory hyperalgesia.

### Inhibition of Rho/ROCK signaling suppresses the activation of pro-inflammatory cytokines by LPS

**Figure 5:**
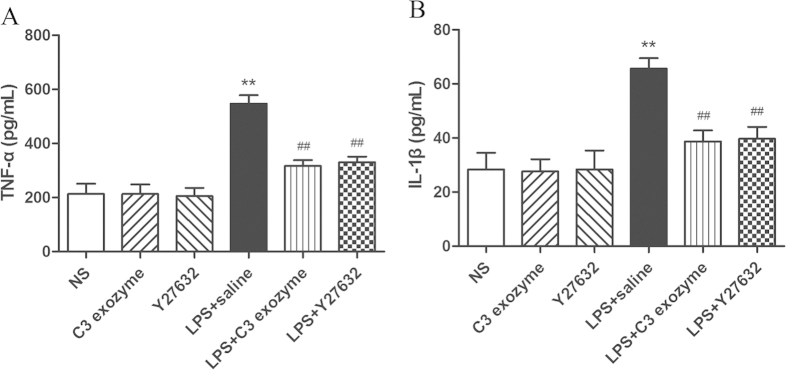
Pro-inflammatory cytokine production induced by LPS depends on Rho/ROCK signaling. Mice received intrathecally-administered C3 exoenzyme or Y27632, followed by LPS 30 min later. At 3 h after LPS injection, the 4–5th lumbar spinal cord was collected for (**A**) TNF-α, (**B**) IL-1β determination by ELISA. **P < 0.01 compared with NS control; ##*P *< 0.01 compared with LPS+saline group. *n *= 8 mice in each group.

To further define the mechanisms by which Rho/ROCK signaling may be associated with LPS-induced inflammatory pain, we examined the effect of suppressing Rho/ROCK activation on the expression of TNF-α and IL-1β in the dorsal horn. The Rho/ROCK pathway inhibitors C3 exoenzyme and Y27632 were delivered intrathecally 30 min prior to intraplantar injection of LPS. Pretreatment with these inhibitors blocked the increase in TNF-α and IL-1β release upon LPS injection (Fig. 5A,B). Taken together, these findings support the idea that activation of Rho/ROCK signaling may induce and maintain inflammatory pain by LPS by regulating pro-inflammatory cytokines, including TNF-α and IL-1β.

## Discussion

Our study reveals a critical role for Rho/ROCK signaling in the production and persistence of inflammatory pain by intraplantar injection of LPS. LPS-inducing pain activates RhoA and ROCK2 signaling, which may contribute to hyperalgesia by regulating the pro-inflammatory cytokines TNF-α and IL-1β in the dorsal horn. The principle findings are 3-fold: (a) LPS treatment-induced inflammatory pain leads to the activation of the RhoA/ROCK pathway, which is associated with a remarkable increase of spinal c-fos protein expression. (b) RhoA/ROCK signaling activation in the dorsal horn causes a rapid increase of TNF-α and IL-1β production. (c) Spinal blockade of the Rho/ROCK signaling pathway inhibits LPS-induced inflammatory hyperalgesia and induction of c-fos, as well as the release of pro-inflammatory cytokines. Taken together, these findings are consistent with the possibility that the application of LPS to the mouse paw induces the release of pro-inflammatory cytokines through the Rho/ROCK signaling pathway, which contributes to inflammatory pain.

LPS is the major molecular component of the outer membrane of Gram-negative bacteria. The presence of this molecule is regarded as a sign of bacterial infection and is responsible for the development of local inflammatory responses. LPS is a well-recognized TLR4 agonist, which leads to the activation of downstream signaling pathways that enhance inflammatory response^29^. Additionally, LPS can activate other intracellular signaling pathways. For example, intratracheal instillation of LPS can induce acute lung injury by activating Rho signaling, and consequently, blocking Rho signaling suppresses LPS-induced lung barrier dysfunction and inflammation *in vivo* and *in vitro*^30^,^31^. Previous work has demonstrated that spinal delivery of LPS induces allodynia^32^,^33^. However, the mechanisms behind the algesic effects of LPS remain unclear. We report here that the Rho/ROCK signaling pathway is activated during LPS-induced hyperalgesia. Intraplantar injection of LPS simultaneously activated RhoA and ROCK at 3 h after treatment, and conversely, spinal blockage of Rho/ROCK signaling with C3 exoenzyme and Y27632 attenuated LPS-induced hyperalgesia. Additionally, paw injection of LPS resulted in marked sensitization of spinal neurons, which is coincident with the activation of spinal c-fos, but pretreatment with C3 exoenzyme and Y27632 at 30 min before the injection of LPS prevented the increase of spinal c-fos expression. These findings provide strong evidence that the Rho/ROCK pathway may be among the important signaling pathways that are activated by LPS, and thus are involved in inflammatory pain development. Because these inhibitors can target other Rho and ROCK pathways, it is also possible that related Rho/ROCK pathways may be involved.

RhoA/ROCK signaling regulates a number of cell functions, including contraction, migration, adhesion, cell cycle progression and cell apoptosis^34^. Moreover, this pathway has been shown to have an important role in a wide variety of pathological states ranging from Alzheimer’s disease^35^, pulmonary hypertension^36^ and acute lung injury mediated by LPS^37^. The RhoA/Rho kinase signaling pathway has also received considerable attention for its involvement in neuroinflammation-induced neuropathic pain^19^,^20^,^38^. Recently, the membrane-localization of RhoA was shown to be increased in the spinal cord of diabetic mice, suggesting that spinal RhoA is activated under diabetic conditions. In addition, several reports have indicated that RhoA/ROCK signaling is involved in hyperalgesia^19^,^20^,^21^. Intrathecal administration of lysophosphatidic acid produces thermal hyperalgesia through activation of the RhoA/ROCK pathway^39^. Thermal hyperalgesia in partial sciatic nerve-ligated animals is blocked by ROCK inhibitor^21^, suggesting that ROCK inhibitor may be efficacious in neuropathic and nociceptive pain models. Collectively, these experimental studies suggest that the RhoA/ROCK signaling pathway plays an important role in the pathogenesis of neuropathic pain. In the present study, we extended the understanding of the role of Rho/ROCK signaling in pain by demonstrating that the spinal Rho/ROCK pathway contributes to LPS-induced inflammatory hyperalgesia. Because LPS is a component of all gram-negative bacteria and its association with inflammatory pain is well characterized, our findings are likely to be applicable to a variety of models of inflammatory pain. Notably, the RhoA/ROCK pathway also has been shown to mediate zymosan-induced paw inflammation in rats^40^. However, the general applicability of our findings is not known. Experiments to assess the role of the Rho/ROCK pathway in carrageenan and CFA-induced hyperalgesia may help to verify the broad applicability of our findings.

In the dorsal horn of the spinal cord we have observed that paw application of LPS induces RhoA/ROCK-dependent production of pro-inflammatory cytokines, including TNF-α and IL-1β. These pro-inflammatory cytokines are known to play an important role in carrageenin-induced mechanical hyperalgesia in mice. Carrageenin induces the production of TNF-α, which in turn induces the production of IL-1β and is responsible for inducing hyperalgesia^41^. Similarly, we found that LPS-induced TNF-α production was initially detectable at 1 h, and peaked at 3 h, while IL-1β production was delayed compared with TNF-α. On the basis of our findings, we speculate that LPS-induced activation of the RhoA/ROCK signaling pathway results in increases in TNF-α and IL-1β production. However, it is not clear whether or not the effects are direct. Though LPS activation in the periphery occurs through its binding to the TLR4 receptor^4^, the spinal activation of Rho/ROCK is presumed to occur through an alternate upstream signaling pathway. Biochemical assays to localize RhoA/ROCK and cytokines in the spinal cord could help to more precisely determine the timing and location of activation. Additionally, increased understanding of the role of RhoA/ROCK may help to identify upstream pathway components and lead to new strategies for pain management.

In conclusion, the present results indicate that LPS-induced hyperalgesia depends on the activation of the Rho/ROCK pathway, leading to increases in the release of pro-inflammatory cytokines, which are responsible for inducing the activation of the dorsal horn neuron. Therefore, developing drugs that selectively target the RhoA/ROCK pathway may improve the management of inflammatory pain. Our study assessing the mechanism of LPS-induced inflammatory pain mainly focused on the RhoA/ROCK pathway; therefore, a role for other Rho GTPases has not been established. For example, it is unclear whether Rac and Cdc42 (the most extensively characterized members of Rho GTPases) mediate the effects of LPS. Therefore, other mechanisms might be involved in LPS-induced inflammatory pain. In the present study, we revealed for the first time that Rho/ROCK signaling pathway-associated inflammatory pain may be caused by bacterial infection. We believe our findings may open a new avenue to understanding the molecular mechanisms of inflammatory hyperalgesia, leading to novel therapeutic strategies for the prevention of inflammatory pain.

## Methods

### Animals, drugs, and drug administration

Adult male C57BL/6 mice (20–25 g) were obtained from the Experimental Animal Center, Medical College of Yangzhou University. Mice were housed under a 12 h light/dark cycle with free access to food and water. The care and treatment of all animals was in strict accordance with protocols approved by the Animal Care and Use Committee of the Medical College of Yangzhou University (Yangzhou, Jiangsu Province, China), and according to the Declaration of the National Institutes of Health Guide for Care and Use of Laboratory Animals (Publication No. 80–23, revised 1996). LPS, C3 exoenzyme and Y27632 were purchased from Sigma (St. Louis, MO, USA) and were resolved in normal saline. In the first series of experiments, mice received intraplantar (e.g. subcutaneous injection in the plantar surface of the paw) injection of LPS (100 ng/paw) or normal saline (20 μL). In the second series of experiments, C3 exoenzyme (10 pg) and Y27632 (10 nmol) were injected intrathecally (L4–L5 spinal level ) in a volume of 5 μL per mouse, 30 min before intraplantar injection of LPS. All drug doses were selected based on previous reports^19^,^20^,^38^ and our preliminary experiments.

### Assessment of pain behavior

Mechanical allodynia was assessed by using electronic von Frey filaments (IITC Life Science Inc., Victory Blvd Woodland Hills, CA). Animals were placed in individual plastic boxes (20 × 25 × 15 cm) on a metal mesh floor and allowed to acclimate for 1 h. The filament was applied to designated loci distributed over the plantar surface of the foot. Brisk withdrawal or paw flinching was considered as a positive response. The paw withdrawal threshold (PWT) was determined by sequentially increasing and decreasing the stimulus strength (the “up- and -down” method), and the data were analyzed using the nonparametric method of Dixon, as described by Chaplan *et al.*^42^.

Thermal hyperalgesia was measured using an IITC Plantar Analgesia Meter (IITC Life Science Inc., Woodland Hills, CA) to measure paw withdrawal latency as described previously^43^. Briefly, each animal was placed in a box containing a smooth, temperature-controlled glass floor. The heat source was focused on a portion of the hindpaw, which was flush against the glass, and a radiant thermal stimulus was delivered to that site. The stimulus was shut off when the hindpaw moved, or after 20 s to prevent tissue damage. The time from the onset of radiant heat to the endpoint was the paw withdrawal latency (PWL). The radiant heat intensity was adjusted to obtain a basal PWL of 10–12 s. Thermal stimuli were delivered 3 times to each hindpaw at 5–6 min intervals.

### Immunohistochemistry

Mice were anesthetized using sodium pentobarbital (60mg/kg, i.p. injection), and perfused intracardially with 0.9% saline followed by 4% formaldehyde. The spinal cord of L4–5 was removed and post-fixed in 4% paraformaldehyde for 3 h at room temperature. It was the equilibrated in 30% sucrose in phosphate buffer overnight at 4 °C. Thirty micrometer transverse series sections were cut on a cryostat and stored in phosphate buffer. For immunofluorescence staining, free-floating sections were blocked in TBS containing 5% donkey serum at room temperature for 2 h, and then incubated in primary antibody at 4 °C overnight. Sections were then washed in PBS (3 times for 5 min each), followed by incubation in secondary antibody at room temperature for 2 h and additional washing. Sections were mounted on slides and covered with 90% glycerin, and then observed under a confocal microscope (FluoView FV1000; Olympus). The anti-c-fos antibody (Abcam) was diluted 1:200. Quantification of immunohistochemical results was done for three sections from spinal cord dorsal horn layer I, II, V, each of which are associated with pain (*n *= 8 mice per group). Sections were taken from the region within lumber segments L3–L5.

### Western blotting

Mice were anesthetized using sodium pentobarbital (60 mg/kg i.p.). The dorsal half of the 4–5th lumbar spinal cord was removed, immediately frozen in liquid nitrogen, and stored at −80 °C. Tissues were sonicated in ice-cold (4 °C) RIPA lysis buffer (Beyotime Institute of Biotechnology) containing a cocktail of protease and phosphatase inhibitors. After incubation on ice for 15 min, homogenates were centrifuged at 12,000 rpm for 20 min at 4 °C. Protein concentrations were measured using a bicinchoninic acid (BCA) Protein Assay Kit (Pierce). Protein samples were then denatured at 95 °C and separated by 8% SDS-PAGE. After transfer to PVDF membranes and blocking with 5% nonfat milk, the membranes were incubated overnight at 4 °C with anti-RhoA (1:500; Abcam), anti-ROCK2 (1:500; Abcam) or anti-β-actin (1:1000; Abcam) primary antibodies. The membranes were washed with wash buffer and incubated for 2 h with alkaline phosphatase-conjugated secondary antibody (1:500; Santa Cruz Biotechnology, Santa Cruz, CA) at room temperature. The immune complexes were then detected using a nitro blue tetrazolium/5-bromo-4-c- hloro-3-indolyl phosphate assay kit (Sigma, St. Louis, MO). Western blots were analyzed using densitometry with Adobe Photoshop software (Adobe Systems Inc.).

### RhoA activity assay

Active GTP-bound RhoA was detected using lysates collected from spinal dorsal horn tissue subjected to pull-down assay with a RhoA activation assay kit (Abcam) according to manufacturer’s indications. Briefly, supernatants were incubated with anti-active RhoA Mouse monoclonal antibody and protein A/G Agarose bead slurry at 4 °C (×1 h) on a rotator. Bead-precipitated proteins were fractionated and immunoblotted with antibody against RhoA.

### ELISA

At the indicated times, animals were terminally anesthetized, and the dorsal half of the 4–5th lumbar spinal cord was collected. The levels of the pro-inflammatory cytokines TNF-α and IL-1β were analyzed using a commercially available ELISA kit specific for mouse TNF-α and IL-1β (R&D Systems, Minneapolis, MN, USA). Briefly, the samples were sonicated in 300 μl of cold Iscove’s culture medium containing 5% fetal calf serum and a cocktail enzyme inhibitor (100 mM amino-n-caproic acid, 10 mM EDTA, 5 mM benzamidine–HCl, and 0.2 mM phenylmethylsulfonyl fluoride). Sonicated samples were centrifuged at 14,000 rpm at 4 °C for 10 min. The supernatants were used to determine the levels of TNF-α and IL-1β. Tissue cytokine concentrations were expressed as ng of protein/mL.

### Statistical analysis

All data were presented as means ± SEM, and differences were considered significant when *P *< 0.05. SPSS Rel 15 (SPSS Inc) was used to perform all statistical analyses. Statistical analysis of two groups was performed using individual Student’s t tests, and statistical analysis of more than two groups was performed using one-way analysis of variance (ANOVA) followed by a Tukey’s post hoc test. Alterations in the behavioral responses to mechanical or thermal stimuli over time among groups were assessed using two-way analysis of variance (ANOVA) with repeated measures followed by Bonferroni’s post-hoc tests.

## Additional Information

**How to cite this article**: Wang, C. *et al.* Inhibition of the Rho/Rho kinase pathway prevents lipopolysaccharide-induced hyperalgesia and the release of TNF-α and IL-1β in the mouse spinal cord. *Sci. Rep.* **5**, 14553; doi: 10.1038/srep14553 (2015).

## Supplementary Material

Supplementary Information
