# Supplementary material for: Inhibition of the Rho/Rho kinase pathway prevents lipopolysaccharide-induced hyperalgesia and the release of TNF-α and IL-1β in the mouse spinal cord
Source: Sci Rep. 2015 Sep 29;5:14553. doi: 10.1038/srep14553 (PMC4586490; doi:10.1038/srep14553)

Title page:

Inhibition of the Rho/Rho kinase pathway prevents lipopolysaccharide-induced hyperalgesia and the release of TNF-α and IL-1β in the mouse spinal cord

Author: Cunjin Wang[1#], Siyuan Song[2#], Yang Zhang[1], Yali Ge[1], Xiangzhi Fang[1], Tianfeng Huang[1], Jin Du[1] and Ju Gao[1]*

1 Clinical Medical College of Yangzhou University & Department of Anesthesiology, Subei People's Hospital of Jiangsu Province, Yangzhou, China

2 Jiangsu Key Laboratory of Anesthesiology & Jiangsu Key Laboratory of Anesthesia and Analgesia Application Technology, Xuzhou Medical College, Xuzhou, China

# These authors contributed equally to this work.

*Corresponding Author: Ju Gao. Address: No.98 Nan Tong Western Road, Yangzhou, Jiangsu, 225001, China. E-mail: gaoju_003@163.com. Tel: +86-0514-87373650.

Supplementary figure legend

Dose-related effects of C3 exoenzyme (A), (B) and Y27632 (C), (D) on the pain behavior in LPS-treated mice. C3 exoenzyme, Y27632 or saline was administered intrathecally before LPS. The mechanical hyperalgesia and thermal hyperalgesia were measured 3h after the treatment with LPS. One-way ANOVA, $^{*}P < 0.05$ compared with saline control, $n = 8$ mice in each group.

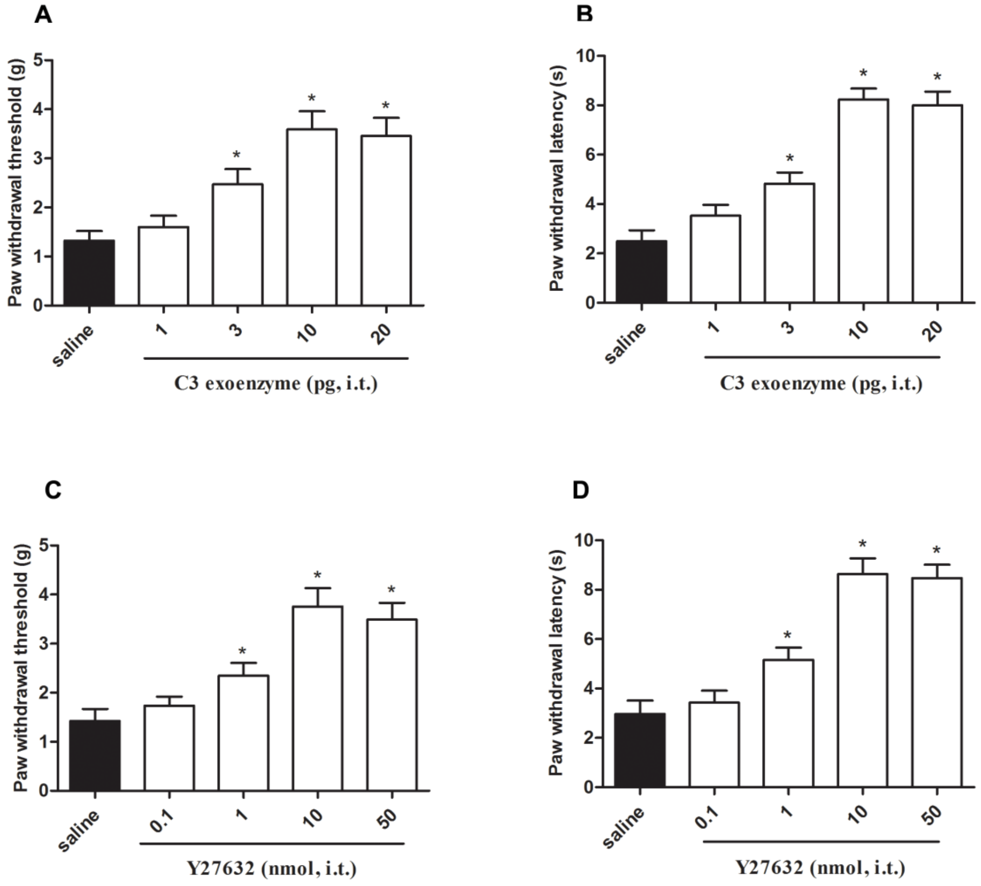

Supplement: Supplementary Information [file srep14553-s1.pdf]
